# Pathogenic *Acanthamoeba castellanii* Secretes the Extracellular Aminopeptidase M20/M25/M40 Family Protein to Target Cells for Phagocytosis by Disruption

**DOI:** 10.3390/molecules22122263

**Published:** 2017-12-18

**Authors:** Jian-Ming Huang, Chen-Chieh Liao, Chung-Ching Kuo, Lih-Ren Chen, Lynn L. H. Huang, Jyh-Wei Shin, Wei-Chen Lin

**Affiliations:** 1 Institute of Basic Medical Sciences, College of Medicine, National Cheng Kung University, Tainan 701, Taiwan; et10005@hotmail.com; 2 Institute of Biotechnology, College of Bioscience and Biotechnology, National Cheng Kung University, Tainan 701, Taiwan; koala6214@gmail.com (C.-C.L.); lrchen@mail.tlri.gov.tw (L.-R.C.); lynn@mail.ncku.edu.tw (L.L.H.H.); 3 Department of Biotechnology and Bioindustry Sciences, College of Bioscience and Biotechnology, National Cheng Kung University, Tainan 701, Taiwan; 4 Department of Microbiology and Immunology, College of Medicine, National Cheng Kung University, Tainan 701, Taiwan; younger811117@gmail.com (C.-C.K.); hippo@mail.ncku.edu.tw (J.-W.S.); 5 Physiology Division, Livestock Research Institute, Council of Agriculture, Taichung 41362, Taiwan; 6 Department of Parasitology, College of Medicine, National Cheng Kung University, Tainan City 701, Taiwan

**Keywords:** *Acanthamoeba*, pathogenesis, M20/M25/M40 superfamily aminopeptidase

## Abstract

*Acanthamoeba* is free-living protist pathogen capable of causing a blinding keratitis and granulomatous encephalitis. However, the mechanisms of *Acanthamoeba* pathogenesis are still not clear. Here, our results show that cells co-cultured with pathogenic *Acanthamoeba* would be spherical and floated, even without contacting the protists. Then, the *Acanthamoeba* protists would contact and engulf these cells. In order to clarify the contact-independent pathogenesis mechanism in *Acanthamoeba*, we collected the *Acanthamoeba*-secreted proteins (Asp) to incubate with cells for identifying the extracellular virulent factors and investigating the cytotoxicity process. The Asps of pathogenic *Acanthamoeba* express protease activity to reactive Leu amino acid in ECM and induce cell-losing adhesion ability. The M20/M25/M40 superfamily aminopeptidase protein (ACA1_264610), an aminopeptidase be found in Asp, is upregulated after *Acanthamoeba* and C6 cell co-culturing for 6 h. Pre-treating the Asp with leucine aminopeptidase inhibitor and the specific antibodies of *Acanthamoeba* M20/M25/M40 superfamily aminopeptidase could reduce the cell damage during Asp and cell co-incubation. These results suggest an important functional role of the *Acanthamoeba* secreted extracellular aminopeptidases in the *Acanthamoeba* pathogenesis process. This study provides information regarding clinically pathogenic isolates to target specific molecules and design combined drugs.

## 1. Introduction

*Acanthamoeba* spp., a free-living amoeba, is found in different environments, including water and soil. In general, *Acanthamoeba* can cause human severe diseases, such as infections of the cornea and the central nervous system [1,2]. Acanthamoeba keratitis (AK) is an infection of the cornea caused by pathogenic *Acanthamoeba* that invades through injuries in the cornea. Most patients wearing contact lenses over a long period of time have a high risk to contract AK [3]. AK may result in lid edema, photophobia, epithelial defects and ring-like stromal infiltrates. Moreover, AK may cause serious consequences, such as permanent visual impairment, if patients are not treated appropriately and immediately [4,5,6,7,8]. However, there is little information that might allow us to comprehend the mechanism by which *Acanthamoeba* causes tissue invasion or infection in these patients. In parasites, the host cell is probably affected by the release of soluble parasite mediators which degrade or otherwise interact with target cells and molecules. In previous studies, researchers found that *Entamoeba histolytica* secretes products (Eh-SEC) or soluble components (Eh-SOL) that selectively damage enteric neurons to cause neurotoxicity [9]. As an example, *E. histolytica* secretes cysteine proteases and EhCP112, which is a protease that overcomes the protective mucus barrier and disrupts cell monolayers to cause invasive intestinal and extra-intestinal infections [10,11]. *Acanthamoeba* is also known to produce serine, cysteine and metalloproteases, and these extracellular protease activities are increased in pathogenic *Acanthamoeba* strains [12,13]. MIP133, a 133 kDa serine protease, has been identified as a principal virulence factor of pathogenic *Acanthamoeba* [14,15]. These studies showed that pathogenic *Acanthamoeba* exhibited diverse secreted proteases and kinases, which could play important roles in infections. However, the pathogenic mechanisms and influences between host cells and excretory-secretory products of *Acanthamoeba* remain undefined.

In our previous study, we reported that datasets of secreted proteomic *Acanthamoeba* strains, including the non-pathogenic ATCC_30010 strain and clinically pathogenic isolates, had been established established [16]. These clinically pathogenic *Acanthamoeba* isolates were collected by National Cheng Kung University (NCKU) Hospital from patients infected with AK [3]. These pathogenic *Acanthamoeba* isolates proved to have high resistance and tolerance to polyhexamethylene biguanide [17]. Furthermore, we investigated the strain-consensus and the pathogenic strain-specific secreted proteins from the comparative analysis for determining the role of these excretory-secretory products. In order to gain a deeper understanding of the contact-independent pathogenesis mechanism of *Acanthamoeba*, this study used an imaging system to investigate the effect on rat glial C6 cells of the *Acanthamoeba* secreted protein (Asp) and live pathogenic *Acanthamoeba* parasites.

Here, we show the cytopathic processes of *Acanthamoeba* and Asp on rat glial C6 cells by time-lapse observation. *Acanthamoeba*-secreted extracellular aminopeptidases play an important role in the *Acanthamoeba* pathogenesis process. Pre-treating the Asp with aminopeptidase inhibitors and specific Acanthamoeba aminopeptidase antibodies could delay the progression of cell disruption and reduce the cell damage during Asp and cell co-incubation.

## 2. Results

### 2.1. Acanthamoeba castellanii Induces Cell Damage in C6 Cells

**Figure 1:**
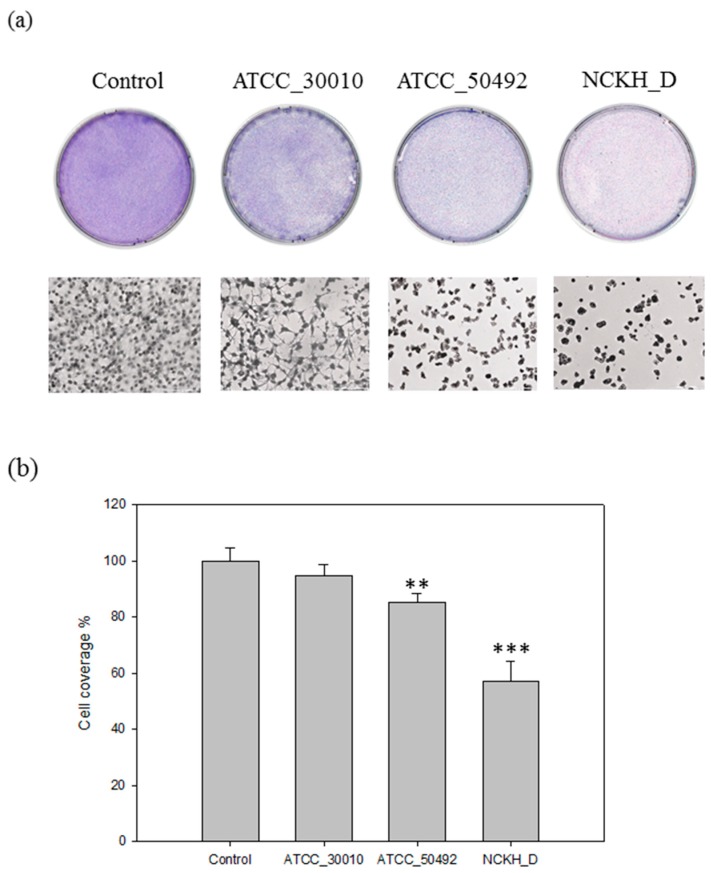
*Acanthamoeba castellanii* induced cell damage of C6 cells. (**a**) Demonstration of effect of different strains *Acanthamoeba castellanii* (ATCC_30010, NCKH_D, ATCC_50492) in the C6 cell line used for the CPE functional assays. The observation of C6 cell co-incubated with *Acanthamoeba castellanii* for 24 h; (**b**) Quantification of the coverage of C6 cells after co-incubated with different strains of *Acanthamoeba castellanii* for 24 h. Those results were means ± SD of triple independent experiments. ** *p* < 0.05, *** *p* < 0.01.

In a previous study, Lagmay et al. established the cytopathic effect (CPE) assay for evaluating and observing the effect of C6 cell co-incubation with different strains of *Acanthamoeba castellani* [18]. Here, in order to clarify the pathogenesis mechanisms of *Acanthamoeba*, we have to choose a suitable strain and the corrected damage-induced duration for observation by CPE assay. Results show that there was no significant change in C6 cell coverage area between C6 cells which were co-incubated with the non-pathogenic ATCC_30010 strain and control (mock) (Figure 1). The live pathogenic *Acanthamoeba* protists, NCKH_D and ATCC_50492, could cause C6 cell damage after co-incubation for 24 h. In our previous study, we have established an RNA-seq database and a comparative proteomic analysis of extracellular secreted proteins, both of them including the ATCC 30010 and the NCKH_D strains [16]. In order to gain a deeper understanding of the pathogenesis mechanism, we focus on the cytopathic effect process of NCKH_D.

### 2.2. Cytopathic Effect Process of Acanthamoeba castellanii

**Figure 2:**
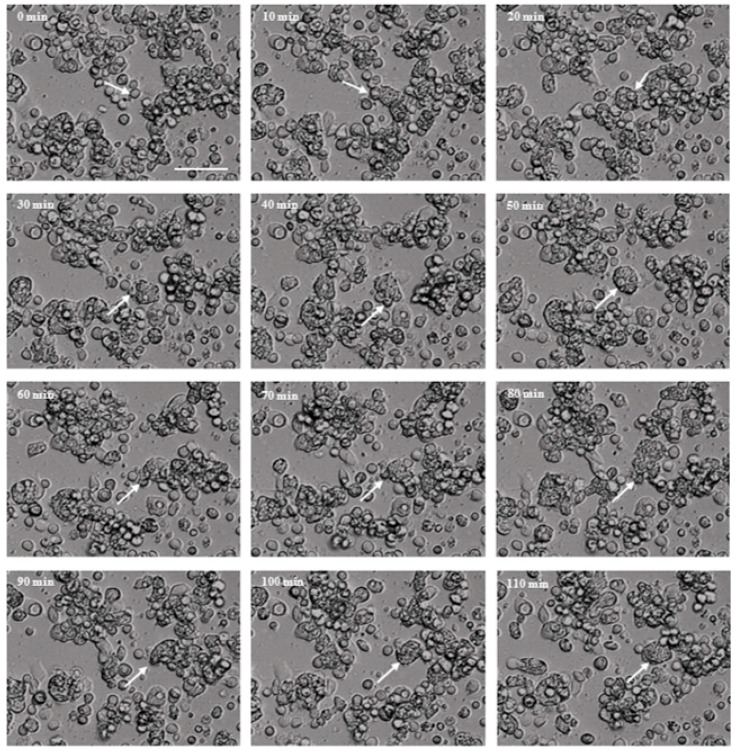
*Acanthamoeba castellanii* NCKH_D induced cell damage by disruption of C6 cells. The observation of C6 cell co-incubated with *Acanthamoeba castellanii* NCKH_D in time-lapse microscopy every 10 min for 2 h. The white arrow: the disrupted C6 cell was attacked by *Acanthamoeba castellanii* NCKH_D. Scale bar = 50 μm.

We observed the cytopathic effect process of NCKH_D-induced C6 cell damage by time-lapse microscopy. Furthermore, we observed that the co-incubated C6 cells would be spherical and floated, even without contacting the protists. Then, the NCKH_D protists attached to the disrupted cells for phagocytosis (Figure 2, supplemental video). From these results, we propose that *Acanthamoeba* releases secreted proteins (Asp) to induce cells to lose adhesion ability and the protists phagocytose these disrupted cells.

### 2.3. Acanthamoeba Secreted Proteins Involves the Co-Cultured Cell Damage Process

**Figure 3:**
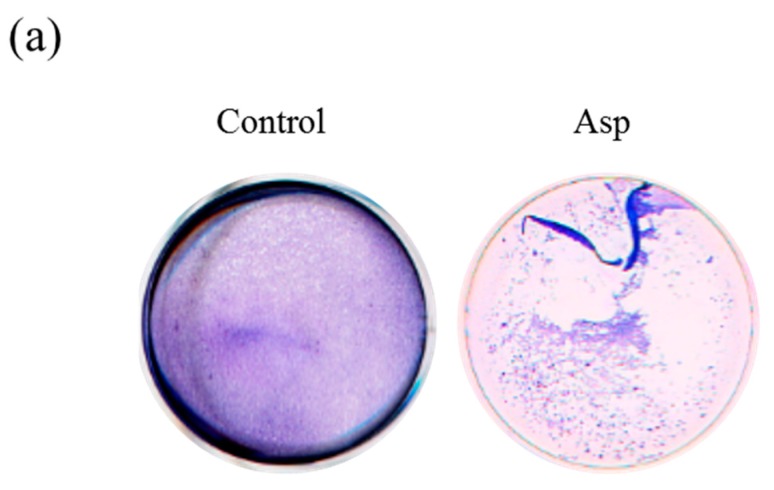

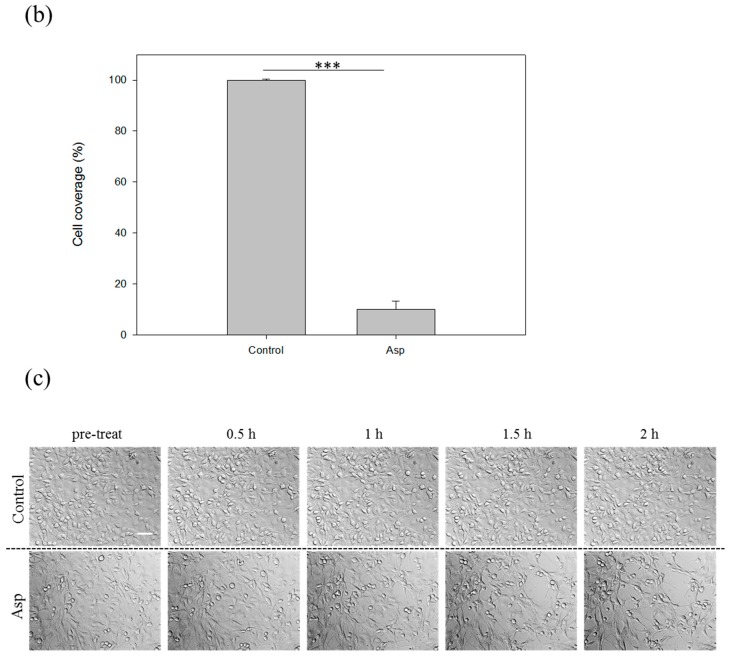
*Acanthamoeba castellanii* NCKH_D secreted proteins induced cell damage by disruption of C6 cells. The observation of C6 cell co-incubated with *Acanthamoeba castellanii* NCKH_D secreted proteins for 2 h by CPE assay (**a**) and time-lapse microscopy (**c**); (**b**) Quantification of the coverage of C6 cells after co-incubated with secreted proteins for 2 h; (**c**) The morphology of C6 cells in control and Asp treated groups by time-lapse microscopy. Those results were means ± S.D. (standard deviation) of triplicate independent experiments. *** *p* < 0.01. Scale bar = 50 μm.

To investigative whether the Asp involved the cell disruption process of *Acanthamoeba*, we isolated the Asp of NCKH_D to treat the C6 cells and observed by CPE assay and time-lapse microscopy. The results of CPE assay were shown that the Asp of NCKH_D could disrupt the co-incubated C6 cells (Figure 3a,b). We observed that the Asp of *Acanthamoeba* NCKH_D induced the cell adhesion losing ability in a time-dependent manner (Figure 3c).

### 2.4. The Biochemical Characterization of Acanthamoeba Secreted Proteins

**Figure 4:**
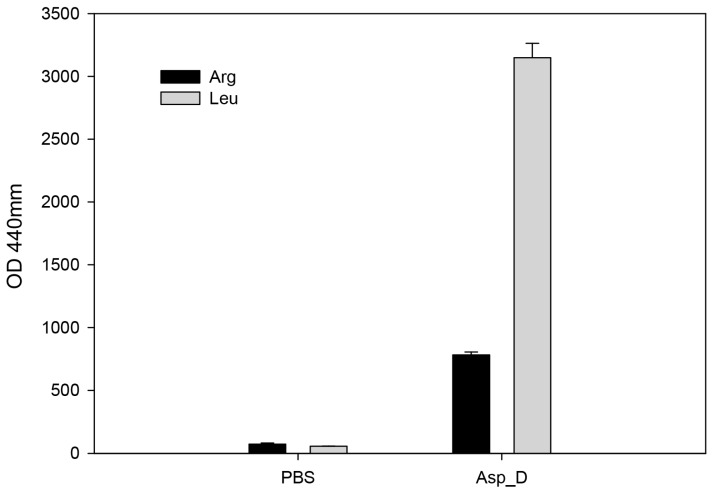
Aminopeptidase activity of *Acanthamoeba castellanii* NCKH_D secreted proteins. Asp_D is Asp from the pathogenic NCKH_D strain.

Cell adhesion mechanism is important for cell migration and cell-cell interactions according to the extracellular matrix (ECM) [19]. The epithelia in animals are surrounded by an extracellular matrix (ECM) of collagen fibers, proteoglycans, as well as multi-adhesive matrix proteins [20]. According to analysis of total protein synthesis in ECM, the amino acid leucine is produced not only in fibronectin but also in collagen [21]. We collected Asp of NCKH_D to treat Leu-AMC and Arg-AMC. Results show that Asp hydrolyzed Leu-AMC rapidly than Arg-AMC, a highly sensitive substrate for aminopeptidase (Figure 4). It suggested that Asp secreted by *Acanthamoeba* may have protease activity to peptides containing the amino acid Leu in ECM to cause cell damage.

### 2.5. The Discovery of M20/M25/M40 Aminopeptidase by Comparative Analysis of RNA-seq

**Figure 5:**
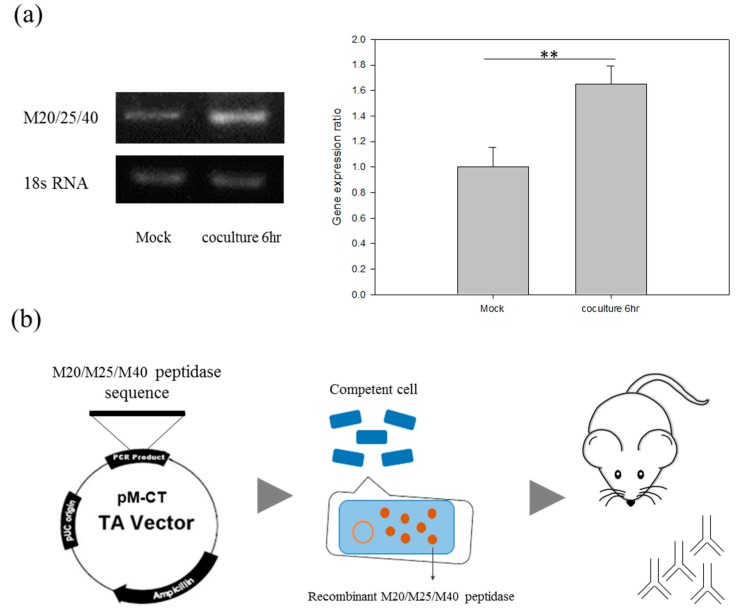

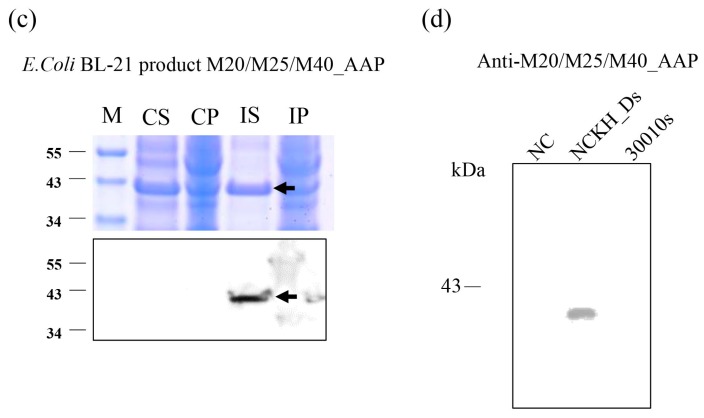
M20/M25/M40 superfamily aminopeptidase gene expression and antibody production. (**a**) Reverse transcription PCR analysis of the M20/25/40 aminopeptidase gene, ACA1_264610, expression level in clinical isolate NCKH_D with or without C6 cells co-incubation. The 18S rRNA expression is used as a control. The production of anti-*Acanthaamoeba* M20/M25/M40 superfamily aminopeptidase antibody; (**b**) The flow chart of anti-*Acanthaamoeba* M20/M25/M40 superfamily aminopeptidase antibody production from recombinant protein to antibody; (**c**) Protein purification and Western blotting of M20/M25/M40 superfamily aminopeptidase recombinant proteins; (**d**) Western blotting of M20/M25/M40 superfamily aminopeptidase polyclonal antibody. M: Marker, CS: Control supernatant, CP: Control pellet, IS: Induce supernatant, IP: Induce pellet, NC: PBS control, NCKH_Ds: NCKH_D secreted protein, 30010s: ATCC_30010 secreted protein. Those results were means ± S.D. (standard deviation) of triplicate independent experiments. ** *p* < 0.05.

In order to clary the virulence factors in Asp, we combined the RNA-seq data and comparative proteomic analysis of extracellular secreted proteins, which were both collected from ATCC 30010 and NCKH_D *Acanthamoeba* cells to identify the candidate proteins [16,17]. From the analysis, we noticed that a protein, the M20/M25/M40 superfamily aminopeptidase protein (ACA1_264610), is expressed and secreted by NCKH_D but not ATCC 30010. Interestingly, we found that the gene expression level of M20/M25/M40 superfamily aminopeptidase protein is upregulated in NCKH_D after *Acanthamoeba* and C6 cell co-culturing for 6 h. The quantified RNA expression patterns of the M20/M25/M40 superfamily aminopeptidase protein are shown in Figure 5a.

To further investigate the function of M20/M25/M40 superfamily aminopeptidase, we prepared an anti-*Acanthamoeba* M20/M25/M40 superfamily aminopeptidase antibody. First, we cloned the M20/M25/M40 superfamily aminopeptidase sequence to the pM-CT vector, then transferred it to the BL-21 *E. coli* strain for recombinant M20/M25/M40 superfamily aminopeptidase production (Figure 5b). The recombinant M20/M25/M40 superfamily aminopeptidase was detected by Coomassie Blue staining and anti-His antibody (Figure 5c). After the protein purification and molecular weight identification, the mouse polyclonal antibody production of M20/M25/M40 superfamily aminopeptidase was peformed by Leadgen Company (Tainan, Taiwan). From the result, we confirmed the specificity of NCKH_D secreted M20/M25/M40 superfamily aminopeptidase but not ATCC 30010 by western blot (Figure 5d).

### 2.6. Aminopeptidase Inhibitor and Antibodies Reduce the Cell Damage

**Figure 6:**
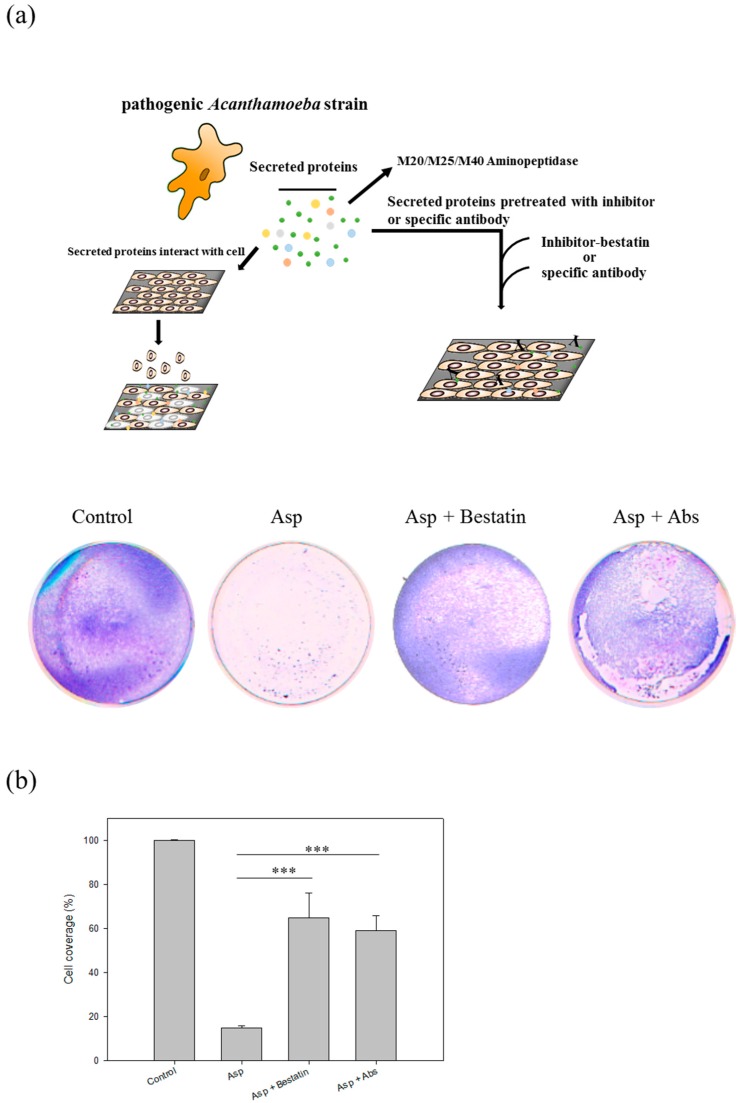
Aminopeptidase of *Acanthamoeba castellanii* induced cell damage of C6 cells. (**a**) Evaluation the treatment effect of inhibitor and antibody of aminopeptidase in Acanthamoeba infection. The inhibitor and antibody reduced the cell disruption of C6 cells co-incubation with Asp for 2 h by CPE functional assays; (**b**) Quantification of the coverage of C6 cells after co-incubated with secreted proteins for 2 h. Bestatin: inhibitor. Abs: the specific antibody of M20/M25/M40 aminopeptidase. Those results were means ± SD of triplicate independent experiments. *** *p* < 0.01.

In our previous study, we noticed that a protein, the M20/M25/M40 superfamily aminopeptidase protein (ACA1_264610), is expressed and secreted by pathogenic *Acanthamoeba castellanii* strain (NCKH_D) but not the non-pathogenic ATCC 30010 strain [16]. To elucidate the importance of M20/M25/M40 superfamily aminopeptidase protein in the contact-independent pathogenesis mechanism of *Acanthamoeba*, we use the aminopeptidase inhibitor, bestatin, a competitive and specific inhibitor of aminopeptidase B, leucine aminopeptidase, and triamino peptidase [22,23], and the specific polyclonal antibodies to pre-treat the Asp of NCKH_D for knockout the aminopeptidase and then co-incubated them with cells. Results show that the cell disruption induced by Asp could be reduced by pre-treating with bestatin (0.42-fold change) and M20/M25/M40 aminopeptidase-specific antibodies (0.49-fold change) (Figure 6). Evaluation the effect of aminopeptidase inhibitor and antibody co-cultured by C6 cells are shown in Figure S1.

## 3. Discussion

The innate immune system is an important strategy to defend the host from infection by other organisms such as pathogens. The first line of defense, the epithelial surfaces forming a physical barrier, prevents most infectious agents from invading organisms. However, pathogens adopt different strategies to invade epithelial barriers and disseminate through the bloodstream to other organs. A previous study showed that the bacterium *Vibrio vulnific* expresses the MARTX toxin to induce intestinal epithelial dysfunction and increase its transmigration [24]. The pathogenesis of *Acanthamoeba* involves parasite-mediated phagocytosis and cytolysis of corneal epithelial cells and gives rise to disease [25]. The previous study showed that the *Acanthamoeba* isolates from AK patients, H-1 and IB-1-7, also produced CPE on rat glial C6 cells in a dose- and time-dependent manner [18]. It was proved that both the trophozoites and the culture cell-free supernatants from pathogenic *Acanthamoeba* strains could produce cytopathic effect on C6 cells. The report suggested a role of *Acanthamoeba*-secreted products as one of the pathogenic mechanisms on the target cells but they did not identify the pathogenic secreted proteins or other factors. Generally, there are contact-dependent and contact-independent mechanisms for host cell injury. In the life cycle of *Acanthamoeba*, the parasite adheres to the host cell when infecting through injury. The previous study showed that mannose-binding protein (MBP) expressed by pathogenic *Acanthamoeba* plays a major role in mediating host and parasite interactions [26]. It induces secondary incidents like interference with host intracellular signaling pathways and phagocytoses host cells by secreting toxins until cell death occurs. Follow this finding, Hurt found that when pathogenic *Acanthamoeba castellanii* trophozoites are grown in methyl-α-d-mannopyranoside, they are induced to secrete a novel 133-kDa protein that is cytolytic to corneal epithelial cells [15]. The secretion of a 133-kDa protein, mannose-induced protein (MIP-133), was significantly upregulated by mannose, but the supernatants from *A. castellanii* grown without mannose did not produce MIP-133. In the initial pathogenic cascade, before the binding, there must be some pathogenic secreted/released factors which play an important role. The purpose of this study was to examine the secretion of cytopathic factors by *A. castellanii*.

The main fibrous ECM proteins which are collagens, elastins, fibronectins, and laminin provide tensile strength or regulate cell adhesion as well as migration [27]. The ECM proteases of some parasites in host cells have recently been reported. The majority of pathogens depend on contact-independent mechanisms to secrete hydrolytic enzymes like elastases [28], phospholipases [29], glycosidases and a variety of serine, cysteine, and metalloproteases for the host cell damage [6,7,25,30]. Amoebic collagenase activity was first described in 1982. It showed that *E. histolytica* produces membrane-bound enzyme to digest collagen type I and type III at neutral pH and 37 °C [31]. Furthermore, *A. polyphaga* binds to the ECM proteins such as collagen type IV, fibronectin and laminin [32]. *Acanthamoeba* has been shown to promote the cleavage of host plasminogen to plasmin activating host matrix metalloproteinases and resulting in degradation of the basement membranes [33]. The previous study has been showed that the secreted protein profiles of non-pathogenic and pathogenic *Acanthamoeba* strains [16]. However, the function of secreted proteins is still unknown.

Here, we observed that *Acanthamoeba* NCKH_D, secretes extracellular aminopeptidases to target cells for phagocytosis by disruption. Therefore, we suggest that it is possible that the disruption of cells via Asp is helpful to digestion by *Acanthamoeba*. In accordance with previous studies, trophozoites interacted with epithelia causing morphological changes and a rapid decrease of resistance and an increased paracellular permeability of epithelial cell layers [34,35,36]. Then, parasites invade dysfunctional epithelial barriers and disseminate through the bloodstream to other organs thus causing serious diseases. Overall, our evidence suggests that the secreted protein of *Acanthamoeba* plays an important role in causing host cell disruption in early steps. This study provides information regarding clinically pathogenic isolates to target specific molecules and design combined drugs.

## 4. Materials and Methods

### 4.1. Culture of Acanthamoeba Protozoa

*Acanthamoeba* species were axenically cultured in a protease peptone-yeast extract-glucose (PYG) medium and antibiotics (50 μg/mL streptomycin and 100 U/mL of penicillin), pH 6.5, at 28 °C in cell culture flasks. Trophozoites were harvested at the logarithmic growth phase after cultivation for 3–5 days. The clinical isolates were isolated from the corneal ulcers of patients who were diagnosed with AK in the Cheng Kung University Hospital [3,17,37].

### 4.2. Assay of Cytopathic Effects (CPE)

Rat C6 cells were plated 3 × 10^5^ in a 24-well plate for incubating with *Acanthamoeba* and secreted proteins. After 2 h co-incubation, the cells were washed, paraformaldehyde-fixed, and then stained with Giemsa stain (Merck, Darmstadt, Germany) [18]. The quantitation of the cells coverage area is used by Image-Pro Plus analysis. The coverage area ratio of co-incubation compared to control.

### 4.3. Live Cell Imaging of C6 Cells Co-Incubation with Acanthamoeba

The live cell image analysis was used for the microscopy (Olympus Cell^R^, Tokyo, Japan). The morphology of rat glial C6 cells was observed after co-incubating with *Acanthamoeba* and their secreted proteins for 24 h.

### 4.4. Isolation of Secreted Proteins

*Acanthamoeba*, at a density of 1.0 × 10^6^ parasites/mL approximately, were washed three times and re-suspended in PBS buffer for 4 h, after the parasites were removed by centrifugation. The cell-free media containing secreted proteins was filtered through Amicon^®^ Ultra-4 Centrifugal Filter Units (Merck Millipore, Darmstadt, Germany) and collected by centrifugation at 959× *g* for 75 min.

### 4.5. Rat Glial C6 Cell Treated with Acanthamoeba and Asp

To find out the probable mechanisms of virulence in vitro, the experiments following the previous study was conducted using a rat glial C6 cell line (ATCC^®^ CCL-107™) to incubate *Acanthamoeba* and secreted proteins [18]. Rat glial C6 cells were cultured in 10 mL Dulbecco’s minimal essential medium (DMEM) supplemented with 10% fetal bovine serum (FBS) and antibiotics (50 μg/mL streptomycin and 100 U/mL of penicillin) at 37 °C incubate with 5% CO_2_. Rat glial C6 cells were cultured 3 × 10^5^ in 24-well plate cell culture dishes. After 24 h of growth, *Acanthamoeba* live cells at a concentration of 3 × 10^5^ were co-cultured with C6 cells. *Acanthamoeba*, at a density of 1.0 × 10^6^ parasites/mL in PBS for 24 h, as well as their secreted proteins were collected. *Acanthamoeba* secreted proteins (150 μg) were co-incubated with rat glial C6 cell for 2 h followed by time-lapse microscopy observations and CPE assays. The effects of 100 μM aminopeptidase inhibitor (bestatin; Sigma-Aldrich, Darmstadt, Germany) and 15 μg antibody of M20/M25/M40 on C6 cell co-incubation with Asp for 2 h were monitored by CPE assay and time-lapse observation.

### 4.6. Biochemical Properties of Asp

Following the steps described in Section 4.4 *Isolation of secreted proteins* and using PBS buffer as the control the activity of Asp was assayed by the hydrolysis of l-leucine-7-amido-4-methylcoumarin hydrochloride (Leu-AMC) and l-arginine-AMC (Arg-AMC, Sigma-Aldrich). Five μL of Asp solution was added to 195 μL of assay buffer (50 mM Tris-HCl, pH 8.0) containing 10 μM Leu-AMC or Arg-AMC and incubated for 30 min at 37 °C. The release of fluorescence was measured at an excitation wavelength of 370 nm and an emission wavelength of 440 nm using a FlexStation 3 Multi-Mode microplate reader (Molecular Devices, Sunnyvale, CA, USA) [23].

### 4.7. Total RNA Isolation

The Total RNA Extraction Miniprep System (VIOGENE) was used. RNA was stored at −70 °C. The entire concentration and A260/A280 ratio of mRNA were measured with ND-1000 (NanoDrop, Thermo, Waltham, MA, USA).

### 4.8. cDNA Synthesis

High Capacity cDNA Reverse Transcription Kits (Applied Biosystems, Foster City, CA, USA) were used in this study. The kit components were allowed to thaw on ice. Reverse transcription conditions were set at the following times and temperatures: 25 °C for 10 min, 37 °C for 120 min, 85 °C for 5 min, finally, the cDNA stored in 4 °C. The reaction volume was set to 20 μL.

### 4.9. Reverse Transcription PCR

One-step reverse transcription PCR was performed with SuperScript™ One-Step RT-PCR with a Platinum^®^ Taq kit (Invitrogen, Waltham, Commonwealth of Massachusetts, USA) to investigate the gene expression of *Acanthamoeba* spp. of M20/M25/M40 superfamily aminopeptidase protein. All of the cDNA was synthesized from 1 μg of total RNA of *Acanthamoeba*. RT-PCR products were separated on the EtBr-stained gel after electrophoresis in 1% agarose. The partial M20/M25/M40 superfamily aminopeptidase forward primer was 5′-TGG ATC GAG TTC AAG GAG GG-3′; and reverse primer was 5′-GCC AGG TGT GCT CGA AGA A GT-3′ with a 364 bp amplification band. 18s rDNA forward primer was 5′-CCC AGA TCG TTT ACC GTG AA-3′ (AcantF900) and reverse primer was 5′-TAA ATA TTA ATG CCC CCA ACT ATC C-3′ (AcantR1100) with a 180 bp amplification band [38]. All experiments were performed independently in triplicate. Image analysis and quantification were performed by using Phoretix 1D (TotalLab, Newcastle upon Tyne, UK).

### 4.10. Protein Analysis

NCKH_D was washed with PAS twice and then scraped and collected. The pellet was incubated with lysis buffer for 5 min at 4 °C. Lysed samples were centrifuged at 15,900× *g* at 4 °C for 5 min. Protein samples were separated with 10% SDS-PAGE (T-Pro, Taipei, Taiwan) were used to separate total proteins. Proteins were then transferred onto the PVDF membranes (Merck Millipore, Darmstadt, Germany). 5% non-fat dry milk in 0.5% PBS-T was used to block the membranes. Immune complexes were formed by incubation of the proteins with our polyclonal antibody against M20/M25/M40 superfamily aminopeptidase overnight at 4 °C. After incubation with primary antibodies, membranes were washed and incubated for 1 h with HRP-tagged secondary antibody (1:10,000; Abcam, Cambridge, UK). Immunoreactive protein bands were detected using Biospectrum 600 Imaging System (UVP, Jena, Germany).

## Supplementary Materials

Supplementary materials are available online.
