# Supplementary material for: Pathogenic Acanthamoeba castellanii Secretes the Extracellular Aminopeptidase M20/M25/M40 Family Protein to Target Cells for Phagocytosis by Disruption
Source: Molecules. 2017 Dec 18;22(12):2263. doi: 10.3390/molecules22122263 (PMC6149796; doi:10.3390/molecules22122263)
Supplement: Supplementary file 1 [file molecules-22-02263-s001.zip › Figure S1.docx]

Pathogenic *Acanthamoeba castellanii* Secretes the Extracellular Aminopeptidase M20/M25/M40 Family Protein to Target Cells for Phagocytosis by Disruption

Jian-Ming Huang ^1,†^, Chen-Chieh Liao ^2,3,†^, Chung-Ching Kuo ^4^, Lih-Ren Chen ^2,5^,
Lynn L. H. Huang ^2,3^, Jyh-Wei Shin ^4,6^ and Wei-Chen Lin ^4,6,^*

^1^ Institute of Basic Medical Sciences, College of Medicine, National Cheng Kung University, Tainan 701, Taiwan; et10005@hotmail.com

^2^ Institute of Biotechnology, College of Bioscience and Biotechnology, National Cheng Kung University, Tainan 701, Taiwan; koala6214@gmail.com (C.-C.L.); lrchen@mail.tlri.gov.tw (L.-R.C.);
lynn@mail.ncku.edu.tw (L.L.H.H.)

^3^ Department of Biotechnology and Bioindustry Sciences, College of Bioscience and Biotechnology, National Cheng Kung University, Tainan 701, Taiwan

^4^ Department of Microbiology and Immunology, College of Medicine, National Cheng Kung University, Tainan 701, Taiwan; younger811117@gmail.com (C.-C.K.); hippo@mail.ncku.edu.tw (J.-W.S.)

^5^ Physiology Division, Livestock Research Institute, Council of Agriculture, Taichung 41362, Taiwan

^6^ Department of Parasitology, College of Medicine, National Cheng Kung University, Tainan City 701, Taiwan

***** Correspondence: wcnikelin@mail.ncku.edu.tw; Tel.: +886-6-235-3535 (ext. 5584);

† The first two authors contributed equally.


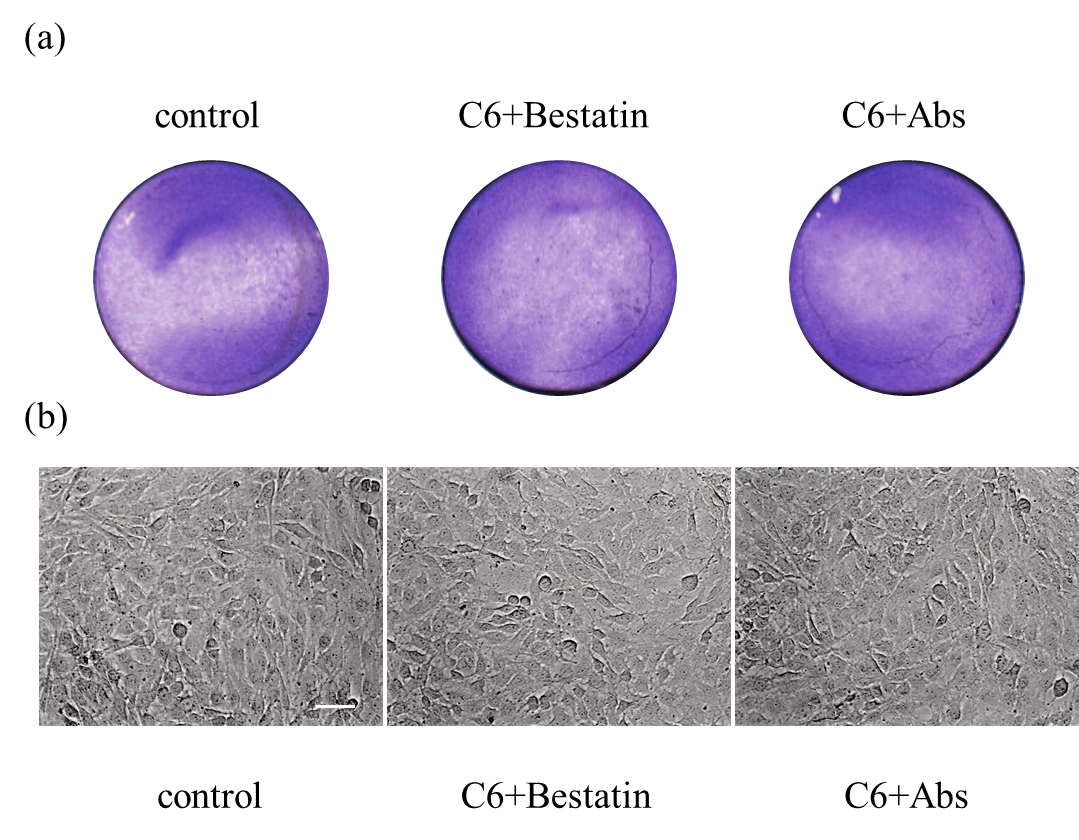


**Figure S1.** Evaluation the effect of aminopeptidase inhibitor and antibody co-cultured by C6 cells. (a) The effect of C6 cells co-cultured with aminopeptidase inhibitor and antibody by cytopathic effect functional assays. (b) The morphology observation of C6 cells co-cultured with aminopeptidase inhibitor and antibody by microscopy. Bestatin: aminopeptidase inhibitor. Abs: the specific antibody of M20/M25/M40 aminopeptidase. Scale bare=50 μm.
